# Enhancing Nursing Care in Monkeypox (Mpox) Patients: Differential Diagnoses, Prevention Measures, and Therapeutic Interventions

**DOI:** 10.7759/cureus.44687

**Published:** 2023-09-04

**Authors:** Tanishq Dubey, Swarupa Chakole, Suyash Agrawal, Anannya Gupta, Pratiksha K Munjewar, Ranjana Sharma, Seema Yelne

**Affiliations:** 1 General Medicine, Jawaharlal Nehru Medical College, Datta Meghe Institute of Higher Education and Research, Wardha, IND; 2 Community Medicine, Jawaharlal Nehru Medical College, Datta Meghe Institute of Higher Education and Research, Wardha, IND; 3 Medicine, Jawaharlal Nehru Medical College, Datta Meghe Institute of Higher Education and Research, Wardha, IND; 4 Internal Medicine, Jawaharlal Nehru Medical College, Datta Meghe Institute of Higher Education and Research, Wardha, IND; 5 Medical Surgical Nursing, Smt. Radhikabai Meghe Memorial College of Nursing, Datta Meghe Institute of Higher Education and Research, Wardha, IND; 6 Nursing, Shalinitai Meghe College of Nursing, Datta Meghe Institute of Higher Education and Research, Wardha, IND

**Keywords:** challenges and future directions, supportive care, infection prevention, therapeutic interventions, prevention and control, differential diagnosis, nursing care, monkeypox

## Abstract

Monkeypox (Mpox), a rare zoonotic viral infection caused by the monkeypox virus, has been gaining attention due to its potential for human-to-human transmission and its clinical resemblance to other poxvirus infections, such as smallpox and chickenpox. Enhancing nursing care for monkeypox patients is imperative to manage and contain its spread effectively. This review analyzes the key aspects of enhancing nursing care in monkeypox patients, focusing on differential diagnoses, prevention measures, and therapeutic interventions.

Differential diagnosis is crucial in terms of the early recognition and management of monkeypox. Given its similarity to other poxvirus infections, a thorough assessment of clinical symptoms, travel history, and exposure to potential reservoir hosts is essential. Nursing professionals play a pivotal role in eliciting comprehensive patient histories and relaying this information to the medical team for accurate diagnosis. Prevention measures constitute a vital component of nursing care in monkeypox management. Implementing stringent infection prevention and control practices, including isolation protocols, personal protective equipment (PPE) usage, and hand hygiene, is imperative to curbing nosocomial transmission. Nurses are at the forefront of enforcing these measures, educating patients, families, and healthcare staff about their significance, and ensuring strict adherence. Therapeutic interventions in monkeypox largely focus on supportive care and symptom management. Nurses occupy a central role in administering antiviral medications, providing wound care for skin lesions, and monitoring patients for potential complications such as secondary bacterial infections. Psychosocial support is equally important, as patients often experience fear and anxiety due to the disease's contagious nature. Nursing professionals offer compassionate care, address patients' emotional needs, and facilitate communication between patients and their families.

Enhancing nursing care for monkeypox entails a multifaceted approach involving differential diagnoses, prevention measures, and therapeutic interventions. Nursing professionals serve as frontline caregivers, pivotal in early diagnosis, effective prevention strategies, and comprehensive patient management. As the global healthcare community faces an influx of emerging infectious diseases, the lessons learned from managing monkeypox can contribute to the creation of a more resilient and responsive nursing workforce capable of effectively addressing future health challenges.

## Introduction and background

Monkeypox (Mpox), a zoonotic viral infection caused by the monkeypox virus, has garnered increasing attention in recent years due to its potential for human-to-human transmission and its clinical resemblance to other poxvirus infections. Originating in central and western Africa, monkeypox was first identified in 1970 and was considered a rare and localized disease [1]. However, sporadic cases and outbreaks have emerged in various parts of the world, challenging healthcare systems and prompting renewed efforts to enhance nursing care strategies. The intricate interplay between clinical presentation, diagnostic challenges, infection control measures, and therapeutic interventions requires a comprehensive review to provide healthcare professionals, particularly nurses, with the knowledge and strategies to manage this evolving threat effectively [2].

Differential diagnosis of monkeypox remains a critical concern due to its overlapping symptomatology with diseases such as smallpox, chickenpox, and other poxvirus infections. The clinical manifestation of skin lesions, fever, and systemic symptoms often necessitates a nuanced evaluation to distinguish monkeypox from its counterparts accurately. With nurses being the primary point of patient contact, their role in eliciting thorough patient histories, assessing clinical symptoms, and conveying critical information to the medical team is pivotal in ensuring timely and accurate diagnosis [3].

The global interconnectedness of communities has heightened monkeypox transmission risk beyond its traditional endemic areas. This has intensified the significance of robust prevention measures, particularly within healthcare settings. As an integral part of healthcare teams, nurses are entrusted with implementing stringent infection prevention and control practices. Their responsibilities encompass educating patients, families, and fellow healthcare professionals about the contagious nature of the disease and ensuring proper utilization of personal protective equipment (PPE) and adherence to isolation protocols [4].

Furthermore, therapeutic interventions in monkeypox management are primarily supportive and focused on alleviating symptoms and preventing complications. Nurses play a central role in administering antiviral medications, providing wound care, and monitoring patients for secondary infections. The psychosocial impact of monkeypox, including patients' emotional distress and the associated stigma, underscores the need for nursing professionals to deliver holistic care that addresses both physical and emotional well-being [4].

As monkeypox continues to pose challenges to healthcare systems worldwide, it is imperative to consolidate current knowledge, experiences, and best practices in nursing care. This review article aims to comprehensively explore the differential diagnosis intricacies, prevention strategies, and therapeutic approaches related to monkeypox management. By shedding light on the pivotal role of nursing professionals in mitigating the impact of this emerging infectious disease, this review highlights the need to foster a resilient and informed healthcare workforce capable of effectively addressing the dynamic landscape of infectious disease threats [5].

This review article aims to comprehensively examine the various aspects of enhancing nursing care in monkeypox patients. This article endeavors to accomplish several objectives through a synthesis of existing literature. Firstly, it provides a thorough background on monkeypox, including its etiology, epidemiology, and modes of transmission, to establish a foundational understanding of the disease. Secondly, it emphasizes the importance of enhancing nursing care in monkeypox patients and its profound impact on patient outcomes, healthcare system preparedness, and public health in general.

## Review

Monkeypox: overview and epidemiology

Monkeypox (Mpox) is a viral disease caused by the monkeypox virus, a member of the orthopoxvirus family [3]. The virus was first identified in 1958 when an outbreak occurred among monkeys kept for research. Monkeypox involves a rash that progresses through different stages, including macules, papules, vesicles, pustules, and crusts. The disease is generally self-limiting but can get severe, particularly in immunocompromised individuals [6].

Global Prevalence and Distribution

The monkeypox virus has two distinct clades: Clade I and Clade II. The typical symptoms of Mpox include the presence of a skin rash or mucosal lesions that can persist for a duration of two to four weeks. Fever, headache, muscle aches, back pain, fatigue, and swollen lymph nodes often accompany these symptoms [6]. Human transmission of Mpox can occur through physical contact with an infected individual, contact with contaminated materials, or contact with infected animals. Laboratory confirmation of Mpox is established by polymerase chain reaction (PCR) testing on skin lesion samples [7].

The primary approach to managing Mpox is through supportive care. In certain circumstances, vaccines and therapeutics developed for smallpox and approved in specific countries may also be utilized for Mpox treatment. Notably, a global outbreak of Mpox occurred in 2022-2023, caused by a strain called Clade IIb [8,9]. Prevention of Mpox primarily involves avoiding physical contact with individuals with the illness. Vaccination can be an effective preventive measure for individuals at risk of Mpox infection. By implementing these preventive measures and utilizing appropriate treatment strategies, the impact of Mpox can be mitigated, and the spread of the disease can be curtailed [10].

Modes of Transmission

Monkeypox is primarily transmitted to humans through direct contact with infected animals, mainly through bites or scratches or by handling their tissues during hunting, preparation, or consumption. The virus can also be transmitted through respiratory droplets from an infected individual or through contact with objects contaminated with the virus, such as bedding or clothing. Additionally, human-to-human transmission can occur, although it is less efficient than primary zoonotic transmission [11].

In healthcare settings, transmission can occur through close contact with infected patients or their bodily fluids and through contaminated surfaces or equipment. This highlights the importance of implementing proper infection prevention and control measures to prevent healthcare-associated infections [12]. Understanding the definition, etiology, global prevalence, distribution, and modes of transmission of Mpox is crucial for healthcare professionals, including nurses, to effectively recognize, manage, and prevent the spread of the disease. This knowledge forms the basis for providing optimal nursing care and implementing appropriate preventive measures for monkeypox patients.

Differential Diagnosis

Distinguishing monkeypox from other dermatological conditions and infectious diseases is paramount for timely and accurate patient management. In the section on differential diagnosis, we delve into the distinctive clinical features of monkeypox at various stages, aiding healthcare providers in making informed diagnostic decisions. By elucidating the nuances that set monkeypox apart from conditions with similar presentations, nurses can contribute to quicker diagnoses and tailored treatment plans [7].

Prevention Measures

Preventing the spread of monkeypox within healthcare settings and communities is imperative. The section on prevention measures in this review explores various preventive strategies, from isolation protocols for infected patients to infection control measures that minimize transmission risk. Emphasis should be placed on proper hygiene, personal protective equipment, and vaccination strategies. We believe this paper will empower nurses to play a pivotal role in curbing the disease's spread by equipping them with comprehensive knowledge of preventive measures [8-9].

Therapeutic Interventions

While no specific antiviral therapy exists for monkeypox, supportive care and symptomatic management are essential treatment components. The section on therapeutic interventions delves into the therapeutic interventions to alleviate symptoms, prevent complications, and promote patient comfort. Moreover, it examines potential avenues for utilizing existing antiviral treatments and immunomodulatory approaches, drawing parallels with strategies employed in related infections. Nurses can enhance patient outcomes and contribute to well-being by outlining these interventions [10-12].

Differential diagnosis of monkeypox

Clinical Presentation and Symptoms

Monkeypox exhibits a diverse range of clinical manifestations that can resemble other similar conditions, making accurate diagnosis challenging. The initial symptoms of monkeypox are nonspecific and may include fever, headache, muscle aches, and fatigue. These symptoms can be easily mistaken for other viral illnesses [13].

**Figure 1:**
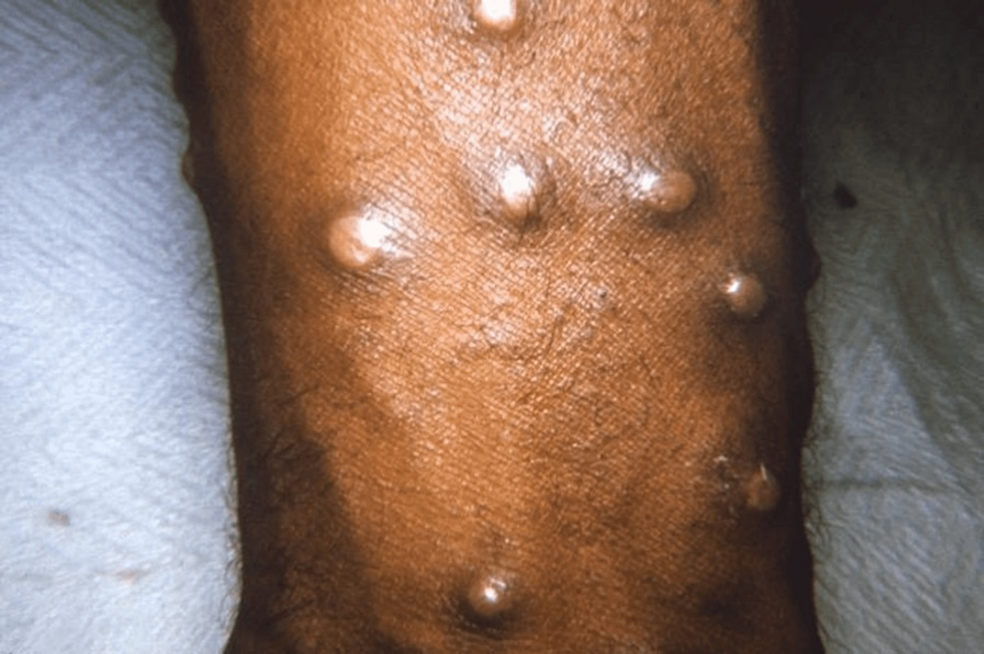
Maculopapular lesions on the arm Open access journal under a CC-BY license. Contributed by Dr. John Noble, Jr., the Centers for Disease Control and Prevention (CDC)

A characteristic rash typically develops within a few days of the onset of symptoms. The rash initially presents as flat, red spots known as macules. Over time, these macules progress into raised bumps called papules. Subsequently, the papules evolve into fluid-filled blisters referred to as vesicles. As the disease progresses, the vesicles may become pustules, characterized by pus-filled contents. Eventually, the pustules develop crusts, leading to the resolution of the rash. It is important to note that the rash can be widespread across the body and may affect various areas, including the face, trunk, and extremities (Figure 1) [14].

In addition to the rash, monkeypox is often associated with swollen lymph nodes (lymphadenopathy). These enlarged lymph nodes are usually tender and can be palpated in regions such as the neck, groin, or axilla [15]. The characteristic rash and associated symptoms of fever, headache, muscle aches, fatigue, and lymphadenopathy can help healthcare professionals suspect monkeypox. However, due to the similarities with other conditions such as chickenpox, smallpox, and certain forms of herpes, laboratory confirmation through diagnostic methods such as PCR, virus isolation, serology, and histopathology is crucial to establish a definitive diagnosis [16]. A thorough understanding of monkeypox's clinical presentation and symptoms is essential for healthcare providers, including nurses, to recognize and differentiate this disease from other similar conditions, facilitating prompt diagnosis and appropriate management.

Distinguishing Monkeypox From Other Similar Conditions

Distinguishing monkeypox from similar conditions is essential for accurate diagnosis and appropriate management. Several diseases share similar clinical features, including smallpox, chickenpox (varicella), and certain forms of herpes [17].

Smallpox: Differentiating between monkeypox and smallpox is essential due to their similarities in rash appearance and progression. While both diseases can manifest with a rash, smallpox typically exhibits a more severe clinical course with more skin lesions than monkeypox. Smallpox lesions are typically concentrated on the face and extremities, whereas monkeypox lesions can occur throughout the body. It is important to note that smallpox has been eradicated, and natural cases no longer occur, whereas monkeypox continues to be a concern [18].

Chickenpox (varicella): Distinguishing monkeypox from chickenpox is crucial as both conditions present with a rash. Chickenpox lesions are often concentrated on the trunk and face, exhibiting a "centripetal" distribution. In contrast, monkeypox lesions tend to be more widespread across the body, with lesions appearing on various regions simultaneously. Monkeypox lesions can progress through different stages simultaneously, including macules, papules, vesicles, pustules, and crusts, while chickenpox lesions tend to be at similar stages of development [19].

Herpes: Certain forms of herpes, such as herpes zoster (shingles), can cause a rash resembling monkeypox lesions. However, there are distinguishing features between the two. Herpes lesions, including those of shingles, are typically localized to a specific dermatome, following the distribution of nerves. In contrast, monkeypox lesions are more diffuse and not limited to a specific nerve distribution pattern. Additionally, the clinical presentation and progression of monkeypox and herpes differ, with monkeypox presenting a distinct evolution of lesions through various stages, as mentioned earlier [20].

Diagnostic Methods and Laboratory Tests

Accurate diagnosis of monkeypox requires laboratory confirmation due to its similarity to other diseases. Diagnostic methods and laboratory tests include:

Polymerase chain reaction (PCR): PCR is a molecular diagnostic technique used to detect the presence of monkeypox viral DNA in patient samples. PCR amplifies specific regions of the viral genome, allowing for virus identification. Samples for PCR testing can include skin swabs, blood, or respiratory secretions. PCR is highly sensitive and specific, providing rapid and accurate results, making it a valuable tool for diagnosing monkeypox [21].

Virus isolation: Virus isolation involves isolating and culturing the monkeypox virus from patient samples in a specialized laboratory. This process requires strict biosafety precautions due to the potential risk of viral transmission. Virus isolation is considered the gold standard for monkeypox diagnosis as it allows for the direct visualization and identification of the virus. However, this method is technically demanding and time-consuming compared to other diagnostic techniques [22].

Serology: Serological tests, such as enzyme-linked immunosorbent assay (ELISA), detect specific antibodies the immune system produces against the monkeypox virus in a patient's blood sample. These tests benefit retrospective diagnosis or epidemiological studies, as they can determine if an individual has been previously exposed to the virus. Serological testing can also aid in confirming the diagnosis of monkeypox in conjunction with other clinical and laboratory findings [23].

Histopathology: Histopathology involves the microscopic examination of skin biopsies taken from monkeypox patients. The biopsied tissue is evaluated for characteristic histopathological changes associated with monkeypox, such as the presence of intracytoplasmic inclusions known as Guarnieri bodies. These inclusions represent viral replication within the cells and are a vital feature of monkeypox. The histopathological examination provides supportive evidence for the diagnosis of monkeypox and can help differentiate it from other similar conditions [24]. Using PCR, virus isolation, serology, and histopathology allows for a comprehensive diagnostic approach to monkeypox. Each method has its own strengths and limitations, and the choice of diagnostic technique may depend on factors such as the availability of resources, laboratory capabilities, and the specific clinical scenario.

Prevention and control measures

Public Health Interventions

Surveillance and reporting: Active surveillance systems are crucial for the early detection and monitoring of monkeypox cases. Healthcare providers and public health authorities can play a crucial role in promptly reporting suspected cases to the appropriate authorities for further investigation and response. This enables tracking of disease patterns, identifying potential outbreaks, and implementing necessary control measures [25].

Contact tracing and isolation: Contact tracing is a fundamental strategy in preventing the spread of monkeypox. It involves identifying and monitoring individuals in close contact with confirmed monkeypox cases. Close contacts are assessed for symptoms and isolated if necessary to prevent further transmission. Isolation measures, such as separating infected individuals from the general population, are vital in containing the spread of monkeypox and reducing the risk of secondary infections [4].

Vaccination programs: Vaccination plays a pivotal role in preventing monkeypox. The smallpox vaccine, which provides cross-protection against monkeypox, can be utilized in regions at risk of monkeypox outbreaks. Vaccination programs should target high-risk populations, including healthcare workers, laboratory personnel, and individuals residing in regions with a history of monkeypox outbreaks. The risk of acquiring and transmitting monkeypox can be significantly reduced by ensuring high vaccine coverage within these populations [26].

Infection Prevention and Control in Healthcare Settings

Personal protective equipment (PPE) protocols: Healthcare workers must strictly adhere to PPE protocols when caring for suspected or confirmed monkeypox cases. This involves wearing gloves, gowns, masks, and eye protection to create a barrier between the healthcare worker and infectious materials. Gloves protect the hands from direct contact with bodily fluids or contaminated surfaces, while gowns provide full-body coverage to prevent contamination of clothing. Masks and eye protection shield the respiratory system and eyes from exposure to respiratory droplets or splashes. Adhering to proper PPE protocols reduces the risk of healthcare workers contracting monkeypox and helps prevent transmission to other patients or individuals in the healthcare setting [27].

Hand hygiene and disinfection measures: Hand hygiene is of utmost importance in preventing the spread of monkeypox. Healthcare workers should practice regular hand hygiene using soap and water or alcohol-based hand sanitizers before and after every patient contact. Thorough handwashing with soap and water for at least 20 seconds is recommended when hands are visibly soiled or when caring for patients with known or suspected monkeypox. Alcohol-based hand sanitizers with at least 60% alcohol content can be used as an alternative when hands are not visibly soiled. Environmental surfaces, equipment, and frequently touched objects should be regularly cleaned and disinfected using appropriate disinfectants effective against the monkeypox virus. Proper disinfection protocols help eliminate the virus from the environment and reduce the risk of transmission [28].

Environmental management: Effective environmental management is critical in reducing monkeypox transmission risk within healthcare settings. This involves several measures, including proper waste management, disinfecting contaminated surfaces, and ensuring adequate ventilation. Healthcare facilities must have appropriate waste disposal systems to safely handle and dispose of contaminated materials. Regular cleaning and disinfection of surfaces, especially high-touch areas, with approved disinfectants, are necessary to prevent the survival and spread of the virus. Adequate ventilation in patient care areas helps maintain clean air circulation and minimizes the concentration of infectious particles. These environmental management practices create a safe and clean healthcare environment, reducing the risk of monkeypox transmission to healthcare workers and other patients [29]. Implementing these prevention and control measures at public health and healthcare setting levels is essential for containing monkeypox outbreaks and protecting healthcare workers and the general population. By adhering to surveillance, contact tracing, vaccination, infection prevention, and environmental management strategies, the spread of monkeypox can be minimized, and the impact of the disease can be reduced.

Therapeutic interventions for monkeypox

Symptomatic Management

Pain management: Pain management is an essential aspect of nursing care for monkeypox patients. Skin lesions and muscle aches can cause significant discomfort and distress. Nonsteroidal anti-inflammatory drugs (NSAIDs) or acetaminophen can be administered to alleviate pain associated with monkeypox. These medications help reduce inflammation and relieve muscle aches and pain [30].

Fever management: Fever is a common symptom in monkeypox cases and can contribute to patient discomfort. Fever control is essential to improve patient comfort and minimize complications associated with high body temperature. Antipyretic medications, such as acetaminophen or ibuprofen, can be administered to reduce fever in monkeypox patients. These medications help lower body temperature and alleviate fever-related symptoms [31].

Pruritus relief: Pruritus, or itching, is a frequent symptom experienced by individuals with monkeypox. It can be distressing and impact the patient's quality of life. To provide relief from pruritus, nurses can employ various strategies. Antihistamines, either oral or topical, can be administered to reduce itching and provide relief. Topical creams or ointments, such as calamine lotion or hydrocortisone cream, can be applied to affected areas to alleviate itching and soothe the skin [32].

Antiviral Therapy

Overview of available antiviral agents: Antiviral medications, such as cidofovir and brincidofovir, have demonstrated efficacy against the monkeypox virus in laboratory studies and have shown some promise in limited clinical experience. These medications belong to nucleoside analogs and work by inhibiting viral replication. Cidofovir has been used off-label to treat severe cases of monkeypox, particularly in immunocompromised individuals or those at high risk of complications. Brincidofovir, a newer antiviral agent, has also shown potential in preclinical studies against monkeypox [33].

Efficacy and limitations: The efficacy of antiviral therapy for monkeypox needs to be well-established due to the limited availability of clinical data. Antiviral agents should be individualized based on the disease's severity, the patient's risk factors, and clinical judgment. Antiviral therapy is typically considered for severe or complicated cases of monkeypox, where the benefits outweigh the potential risks [34].

Supportive Care

Fluid and electrolyte management: Adequate fluid intake is vital for monkeypox patients to maintain hydration and support proper physiological function. In severe cases where oral intake may be insufficient or compromised, intravenous fluids may be necessary to ensure adequate hydration. Closely monitoring electrolyte levels, particularly potassium, is essential to prevent imbalances and ensure optimal patient care [35].

Nutritional support: Proper nutrition is critical in supporting recovery and immune function in monkeypox patients. A well-balanced diet that includes foods rich in vitamins, minerals, and essential nutrients should be provided. This supports the body's ability to heal and fight off infections. In severe cases where oral intake may be challenging or inadequate, enteral or parenteral nutrition may be necessary to meet the patient's nutritional needs and support their overall well-being [36].

Psychological support: The impact of monkeypox can extend beyond the physical symptoms and have psychological effects on patients. The distress and anxiety associated with the disease can significantly affect their emotional well-being. Therefore, providing psychological support is crucial. This may involve counseling sessions to address fears, anxieties, and mental health concerns. Offering reassurance, empathy, and active listening can help alleviate distress and promote overall well-being for monkeypox patients [37].

The therapeutic interventions for monkeypox primarily focus on symptomatic management, antiviral therapy (when indicated), and supportive care. It is essential for healthcare professionals, including nurses, to provide comprehensive care addressing pain, fever, pruritus, fluid/electrolyte imbalances, nutrition, and psychological well-being. Individualized treatment plans should be developed based on the severity of the disease, patient characteristics, and available resources. Close monitoring and evaluation of treatment responses are necessary to optimize patient outcomes [38].

Nursing care strategies for monkeypox patients

Patient Assessment and Monitoring

Regular vital signs monitoring: Nurses play a crucial role in monitoring and documenting the patient's vital signs, including temperature, heart rate, respiratory rate, and blood pressure. This monitoring allows for ongoing assessment of the patient's physiological status and helps evaluate the disease’s progression. Vital signs can provide valuable information about the patient's overall health, response to treatment, and the presence of any complications. Any significant deviations from the normal range can alert the nurse to potential issues requiring immediate attention [39].

Skin assessment: Monkeypox is characterized by skin lesions progressing through different stages. Regular and thorough skin assessments by nurses are essential to monitor the appearance and progression of these lesions. Nurses examine the skin lesions' size, color, texture, and distribution. Additionally, they assess for signs of infection, such as redness, swelling, warmth, or purulent discharge. By closely monitoring the skin lesions, nurses can promptly identify any changes or signs of complications, enabling timely intervention and appropriate wound care management [40].

Pain assessment: Pain is a common symptom experienced by monkeypox patients, mainly due to skin lesions and associated inflammation. Nurses use appropriate pain assessment tools to evaluate the intensity, location, and characteristics of the patient's pain. They also assess the impact of pain on the patient's overall well-being and functional ability. Based on the assessment findings, nurses develop a tailored pain management plan, including administering analgesic medications, applying topical agents, or non-pharmacological techniques such as positioning, relaxation, or distraction. Regular pain assessments allow nurses to monitor the effectiveness of pain management interventions and make adjustments as needed to ensure optimal pain relief and promote patient comfort and well-being [41].

Infection Prevention Strategies

Strict adherence to standard precautions: Nurses should prioritize strict adherence to standard precautions when caring for patients with monkeypox. This includes meticulous hand hygiene practices, such as thorough handwashing with soap and water or using alcohol-based hand sanitizers before and after patient contact. Proper use of PPE is crucial, including wearing gloves, gowns, masks, and eye protection, as appropriate, to minimize the risk of exposure to infectious materials. Nurses should also ensure the disposal of contaminated materials, such as used gloves, masks, and other disposable items, in designated containers [42].

Isolation precautions: Nurses are vital in implementing appropriate isolation precautions for suspected or confirmed monkeypox patients. These precautions are based on transmission-based precautions, which include contact and droplet precautions. Nurses should ensure patients are placed in appropriate isolation rooms or designated areas meeting infection control standards. Contact precautions involve using gloves and gowns when in close contact with the patient or their immediate environment to prevent direct contact transmission. Droplet-related precautions require wearing a surgical mask when in close proximity to the patient to minimize the risk of respiratory droplet transmission. Nurses should diligently adhere to these precautions to minimize the risk of transmission to healthcare workers and other patients [43].

Environmental cleaning and disinfection: Nurses should collaborate closely with environmental services to ensure effective cleaning and disinfection of patient care areas, equipment, and surfaces. Regular and thorough cleaning using appropriate disinfectants helps minimize monkeypox’s spread. Special attention should be given to frequently touched surfaces, such as door handles, bed rails, and medical equipment. All cleaning procedures should adhere to established infection control guidelines and protocols. Nurses should actively communicate with environmental services to ensure that proper cleaning and disinfection practices are implemented consistently throughout the healthcare setting, creating a safe environment for patients and healthcare workers [44].

Psychosocial Support and Patient Education

Emotional support: Nurses are critical in providing emotional support to monkeypox patients, who may experience fear, anxiety, and social isolation due to their diagnosis. By actively listening to their concerns, demonstrating empathy, and utilizing therapeutic communication techniques, nurses can create a supportive environment that helps alleviate distress. This may involve providing a safe space for patients to express their feelings, addressing their worries and fears, and offering reassurance. Nurses can also collaborate with mental health professionals to provide additional psychological support. By attending to the emotional well-being of monkeypox patients, nurses contribute to their overall holistic care and help promote a sense of security and well-being during a challenging time [45].

Patient education: Nurses are crucial in educating monkeypox patients and their families about various aspects of the disease. This includes information on the transmission of monkeypox, preventive measures to minimize the risk of further spread, and the importance of adhering to prescribed medications and treatment plans. Nurses should offer clear instructions regarding wound care, emphasizing proper hygiene practices to prevent secondary infections and complications. Additionally, nurses should educate patients and their families on the significance of isolation precautions, such as limiting physical contact with others and practicing proper hand hygiene, to prevent transmission to others. Patient education equips individuals with knowledge, thereby enabling them to actively participate in their care and take necessary steps to prevent the further spread of the disease. Through effective education, nurses promote health literacy, enabling patients to make informed decisions and engage in behaviors that support their recovery and protect others from infection [7].

Collaborative Multidisciplinary Care

Collaborative care planning: Nurses play a pivotal role in collaborating with various healthcare professionals to develop a comprehensive care plan for monkeypox patients. This collaboration involves working closely with physicians, infection control specialists, and allied health professionals, such as physical and respiratory therapists, to address all patient care aspects. By pooling their expertise and knowledge, the healthcare team can create a tailored care plan that considers the patient’s needs, including their physical, psychological, and social well-being. Regular interdisciplinary team meetings provide an opportunity for effective communication, information sharing, and care coordination. This collaborative approach helps to ensure that all healthcare professionals are on the same page, working towards common goals and providing consistent and coordinated care to monkeypox patients [9].

Referral and follow-up: Nurses are responsible for facilitating timely referrals to specialists when additional expertise or specialized care is required. In the case of monkeypox, referrals may be made to infectious disease experts or mental health professionals, among others, depending on the patient’s specific needs. Infectious disease experts can provide specialized knowledge and guidance on managing monkeypox, including antiviral therapies or complications that may arise. Mental health professionals can offer support and intervention for patients experiencing psychological distress or anxiety related to their diagnosis or treatment. Nurses are crucial in coordinating these referrals, ensuring they are made promptly and the patient receives the necessary care promptly [46]. Nurses have a pivotal role in providing holistic care to monkeypox patients. Through patient assessment and monitoring, infection prevention strategies, psychosocial support and patient education, and collaboration with multidisciplinary teams, nurses can enhance the quality of care, promote patient well-being, and contribute to positive patient outcomes.

Challenges and future directions

Challenges Faced in Enhancing Nursing Care for Monkeypox Patients

Limited resources: Providing optimal nursing care for monkeypox patients can be challenging in resource-limited settings. Adequate availability of necessary equipment, medications, and supplies can help deliver comprehensive care. The challenges may include a lack of isolation facilities, PPE, diagnostic tools, and therapeutic resources. Additionally, limited staffing levels can strain healthcare professionals, affecting their capacity to provide individualized care and implement adequate infection control measures [47].

Inadequate training and education: Nursing professionals may have limited exposure to monkeypox during their education and training, leading to a lack of familiarity with the disease. As a result, there may be challenges in accurately diagnosing monkeypox cases and implementing appropriate management strategies. This includes recognizing the characteristic rash progression, understanding the differential diagnosis, and staying updated with the latest guidelines and treatment options. Insufficient training can impact the quality of care, highlighting the need for continuous education and professional development to enhance nursing competence in managing monkeypox [48].

Psychosocial impact: Monkeypox can have a significant psychosocial impact on affected individuals. Patients may experience fear, anxiety, and social stigma due to the contagious nature of the disease and the physical appearance of the rash. Addressing these psychosocial needs can be challenging for nurses who may require additional training and support in providing appropriate psychological support and counseling. Developing effective communication skills, practicing empathy, and promoting a supportive environment are crucial in addressing the emotional well-being of monkeypox patients. Collaborating with mental health professionals and incorporating psychosocial support into nursing care plans can enhance patient outcomes and overall satisfaction [49].

Research Gaps and Areas for Further Investigation

Antiviral therapy: The current knowledge regarding the efficacy, safety, and optimal dosing of antiviral agents for monkeypox treatment is limited. Further research, including well-designed clinical trials and studies, is needed to evaluate the effectiveness of antiviral therapy in monkeypox patients. By conducting rigorous research, more evidence-based guidelines can be developed to guide healthcare professionals in the appropriate use of antiviral medications, leading to improved treatment outcomes for monkeypox [50].

Long-term effects and complications: Understanding monkeypox's long-term sequelae and complications is crucial for comprehensive patient care. Research focusing on the potential long-term impacts on physical, mental, and psychosocial well-being can provide valuable insights into the needs of monkeypox survivors. Investigating the disease's potential cognitive, emotional, and functional consequences can contribute to developing targeted interventions and support services to enhance the quality of life for those affected by monkeypox [47].

Prevention strategies: Developing effective prevention strategies is essential to reduce the burden of monkeypox and prevent outbreaks. Research efforts should improve existing vaccines, explore new vaccine candidates, and optimize targeted vaccination programs. Additionally, studying the effectiveness of public health interventions, such as surveillance systems, contact tracing, and community education campaigns, can inform the development of evidence-based prevention strategies. By investing in research on prevention strategies, healthcare systems can be better prepared to control monkeypox and mitigate its impact on public health [51].

Innovations in Nursing Practice for Improved Care

Technology integration: Integrating technology, such as telehealth and electronic health records (EHRs), can significantly improve the management of monkeypox cases. Telehealth enables remote consultations, allowing healthcare providers to assess and guide patients without direct physical contact. This can be particularly useful when patients are geographically distant or during public health emergencies. EHRs streamline the documentation and sharing of patient information, ensuring that healthcare professionals can access comprehensive and up-to-date medical data and facilitating coordinated care [52].

Simulation and training: Incorporating simulation-based training and educational programs specific to monkeypox can significantly enhance nurses' knowledge, skills, and preparedness in managing cases. Scenario-based simulations allow nurses to practice recognizing, diagnosing, and managing monkeypox cases in a controlled environment. These simulations provide opportunities to refine clinical judgment, decision-making skills, and effective communication within interdisciplinary teams. Interactive workshops can further supplement theoretical knowledge and promote critical thinking in addressing complex scenarios related to monkeypox care [53]. 

Interprofessional collaboration: Emphasizing interprofessional collaboration and teamwork among healthcare professionals involved in monkeypox care is essential for comprehensive and effective management. Regular interdisciplinary meetings and joint training sessions facilitate care coordination, information sharing, and mutual understanding of roles and responsibilities. This collaborative approach ensures that all healthcare providers, including nurses, physicians, laboratory specialists, and public health experts, work together synergistically to address the diverse needs of monkeypox patients. Interprofessional collaboration improves patient outcomes and enhances decision-making and a more holistic approach to care delivery by fostering effective communication and interdisciplinary cooperation [54]. By addressing the challenges faced in enhancing nursing care, identifying research gaps, and embracing innovations in nursing practice, healthcare systems can strive to improve the care and outcomes among monkeypox patients. Collaboration between researchers, healthcare providers, policymakers, and nursing organizations is essential to drive advancements and implement evidence-based strategies for better prevention, management, and patient support in monkeypox.

## Conclusions

It is evident that tailoring nursing care to the specific needs of monkeypox patients is not only vital but also directly correlated with the proficient management and containment of the disease. The multifaceted role of nurses encompasses early identification and differential diagnosis, meticulous implementation of infection prevention protocols, and the administration of comprehensive therapeutic regimens. Through diligent patient evaluation, stringent infection control practices, provision of psychosocial solace, and active engagement within interdisciplinary teams, nurses significantly contribute to enhancing patient well-being, minimizing transmission hazards, and ultimately, improving outcomes. Nonetheless, optimizing nursing care mandates a dedicated allocation of resources and a robust training framework. Sustained progress and elevated care standards hinge on persistent research endeavors, innovative methodologies, and seamless interprofessional cooperation. The advancement of this field depends on acknowledging the distinctive challenges posed by monkeypox and striving for continuous enhancement in the health prospects of those affected. By bestowing due emphasis and investment into nursing care, we can effectively tackle the intricacies of monkeypox and improve the overall health trajectory of afflicted individuals.
